# Hydroxyl Radical Scavenging by Aucubin: A Mechanistic Study

**DOI:** 10.3390/antiox14111342

**Published:** 2025-11-07

**Authors:** Kunzhe Jiang, Jingran Wang, Wang Yang, Ying Xiong, Meiling Chen, Qiang Zhou, Yanhong Wang

**Affiliations:** 1 Key Laboratory of Rare-Scattered Elements of Liaoning Province, College of Chemistry, Liaoning University, Shenyang 110036, China; 4032332240@smail.lnu.edu.cn; 2 Institute of Applied Ecology, Chinese Academy of Sciences, Shenyang 110016, China; wangjr@iae.ac.cn (J.W.); 15802486105@163.com (W.Y.); m15709611692@163.com (M.C.); zhouqiang@iae.ac.cn (Q.Z.); 3 Shenyang Key Laboratory of Food Safety Testing and Control Technology of Shenyang, Shenyang 110016, China

**Keywords:** aucubin, antioxidant, hydroxyl radical, DFT

## Abstract

This study investigates the antioxidant properties of aucubin (AU), an iridoid compound, focusing on its ability to scavenge hydroxyl radicals (^•^OH) through its hydroxyl functional groups. Gaussian software was employed to model and validate the underlying antioxidant reaction mechanisms. Three primary pathways were examined: hydrogen atom transfer (HAT), sequential electron transfer-proton transfer (SET-PT), and sequential proton loss–electron transfer (SPLET). All calculations were performed using the M06-2X functional within density functional theory (DFT) at the def2-TZVP level, incorporating Grimme’s D3 dispersion correction and the implicit solvation model based on solute electron density (SMD) for water. Various thermodynamic parameters were determined to analyze and compare the antioxidant reactions, including the O-H bond dissociation energy (BDE), ionization potential (IP), proton dissociation enthalpy (PDE), electron transfer enthalpy (ETE), and proton affinity (PA) of the hydroxy groups. The results indicated that the HAT mechanism is the dominant pathway in the scavenging of ^•^OH radicals by AU. The key active sites were identified as the 6-OH group in the aglycone structure and the 6′-OH group in the sugar moiety. Moreover, the polar aqueous environment promoted O-H bond homolysis to enhance the antioxidant activity.

## 1. Introduction

Free radicals are atoms, molecules, or ions that contain unpaired electrons, which render them highly unstable and extremely reactive toward other chemical species. Among them, oxygen-centered free radicals—such as hydroxyl radicals (^•^OH), peroxyl radicals (^•^OOR), and alkoxyl radicals (^•^OR)—are the most reactive. These radicals primarily initiate chain-oxidation reactions through hydrogen abstraction or addition processes, targeting biomolecules such as lipids (especially at double bonds and allylic positions), protein sulfhydryl groups, and nucleic acid bases. Numerous studies have demonstrated that oxidative stress, resulting from an excessive accumulation of oxygen-derived free radicals, is directly associated with more than 200 human diseases, including Alzheimer’s disease, atherosclerosis, and diabetic complications [1]. Therefore, investigating the molecular orbital interactions of specific bioactive components in foods and medicinal plants, as well as elucidating the mechanistic pathways of their active sites, is essential for the rational design of targeted antioxidant therapies.

**Figure 1:**
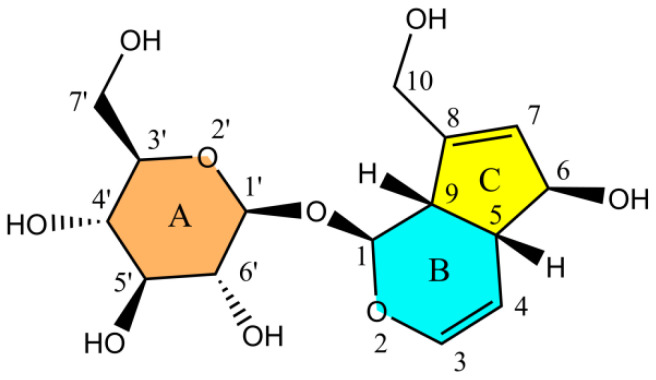
Molecular structure of AU.

Iridoid compounds, important natural antioxidants found in plants, exhibit a broad spectrum of pharmacological activities, including antioxidant, anti-inflammatory, and anticancer effects [2,3]. However, compared to flavonoids and polyphenols, they have been relatively less explored. Aucubin (AU) (Figure 1), a representative iridoid glycoside, is widely distributed in medicinal plants such as *Eucommia ulmoides*, *Scrophularia ningpoensis*, and *Plantago asiatica*. Research on the pharmacological activities of AU has progressed beyond its conventional role as an antioxidant that mitigates oxidative stress, extending into several emerging therapeutic areas. These include its hypoglycemic effects through activation of the IRS/Akt signaling pathway to improve insulin resistance, anti-inflammatory effects by inhibiting the release of IL-6/TN-α inflammatory markers, and cardiovascular protection by preventing the oxidative modification of low-density lipoproteins [4]. Collectively, these findings underscore the potential of AU as a multi-target therapeutic agent for chronic diseases. Furthermore, the antioxidant properties of AU have been successfully applied in commercial products. In the food industry, AU exhibits a half-maximal inhibitory concentration (IC_50_) of 49.20 mg/mL against DPPH free radicals [5], while in skincare formulations, AU has been shown to reduce oxidative damage to macroscopic skin texture by approximately 3% after four weeks of treatment [6]. These applications highlight the promising potential of AU for further pharmaceutical and industrial development. However, most existing research on aucubin (AU) has primarily focused on verifying its general biological activities and optimizing extraction procedures from medicinal plants [7]. In contrast, relatively few studies have specifically investigated its antioxidant mechanisms. Most of the existing work has concentrated on the functional groups within the aglycone moiety (rings B and C), whereas the sugar moiety (ring A) has received limited attention. Moreover, systematic molecular-level investigations into AU’s mechanisms—such as its electron transfer pathways and free radical scavenging sites—remain scarce. This knowledge gap restricts progress in areas such as structural modification, targeted delivery systems, and the development of AU-based functional formulations. Given that the density functional theory (DFT) method generally provides lower computational error than the Hartree–Fock (HF) method when exploring chemical reaction mechanisms [8], DFT has become the preferred theoretical approach for elucidating antioxidant reaction mechanisms in both metal/non-metal-doped materials [9,10] and organic molecules [11]. Therefore, this study employs DFT to elucidate the antioxidant mechanism of AU, identify its predominant reaction pathways and active sites, and establish a molecular-level foundation for its further development and application.

## 2. Assessment of Antioxidant Capacity

### 2.1. Experimental Principle

Following the methodological framework established by Yamaguchi [12], Chen [13], and Zhao et al. [14], hydroxyl radicals (^•^OH) were generated via the Fenton reaction (Fe^2+^ + H_2_O_2_ → Fe^3+^ + ^•^OH + OH^−^). Salicylic acid was introduced into the reaction system to rapidly react with ^•^OH, producing 2,3-dihydroxybenzoic acid, which exhibits a characteristic absorption peak at 510 nm. Aucubin (AU), with its ^•^OH-scavenging activity, competes with salicylic acid for ^•^OH, thereby decreasing the formation of 2,3-dihydroxybenzoic acid and resulting in a reduction in absorbance at 510 nm. The ^•^OH scavenging rate of AU was calculated according to the following equation: Scavenging rate (%) = [(A_0_ − A_x_)/A_0_] × 100%.

(A_0_ represents the absorbance of the blank control group and A_x_ represents the absorbance of the sample group or positive control group.)

### 2.2. Experimental Details

#### 2.2.1. Experimental Materials

Analytical-grade reagents, including aucubin (AU), ferrous sulfate heptahydrate (FeSO_4_·7H_2_O), disodium ethylenediaminetetraacetate dihydrate (EDTA-2Na), salicylic acid, ascorbic acid, hydrogen peroxide (H_2_O_2_), and phosphate-buffered saline (PBS), were purchased from Shanghai Macklin Biochemical Co., Ltd. (Shanghai, China). Ultrapure water was produced using a Milli-Q water purification system (Merck KGaA, Darmstadt, Germany).

#### 2.2.2. Experimental Design

To evaluate the hydroxyl radical (^•^OH) scavenging activity of aucubin (AU), five concentrations were tested: 5.89 mmol/L (A), 2.95 mmol/L (B), 1.47 mmol/L (C), 0.74 mmol/L (D), and 0.37 mmol/L (E). Ascorbic acid at 5.89 mmol/L was used as the positive control, while a blank control (A_0_) without any antioxidant was also included. All experiments were performed in triplicate. The prepared solutions were stored at 4 °C under a nitrogen atmosphere.

#### 2.2.3. Determination of Hydroxyl Radical Scavenging Rate

The hydroxyl radical (^•^OH) scavenging rate was determined at 510 nm using a UV-visible spectrophotometer (Shanghai Mapada Instruments Co., Ltd., Shanghai, China). In a quartz cuvette, 1.0 mL of FeSO_4_-EDTA-2Na solution (60 mmol/L), 1.0 mL of salicylic acid solution (75 mmol/L), and 1.0 mL of AU or ascorbic acid solution were added sequentially. For the blank control (A_0_), 1.0 mL of ultrapure water was used instead. Then, 1.0 mL of H_2_O_2_ solution (90 mmol/L) was quickly added, the cuvette was immediately capped, and the mixture was vortexed for 5 s. The absorbance was recorded within 25 s after vortexing.

#### 2.2.4. Analysis of Test Results

The ^•^OH scavenging rates of AU at different concentrations ranged from 19.44% to 47.04% (Table 1). Compared with ascorbic acid (45.14%), a commonly used antioxidant at the same concentration, AU exhibited a slightly higher scavenging rate (47.04%). These results indicate that AU possesses strong hydroxyl radical scavenging activity, underscoring its potential application in antioxidant research.

## 3. Theoretical Calculation

### 3.1. Calculation Method

All calculations were performed using Gaussian 16 (Version C.01) [15]. Owing to the proven accuracy of the M06-2X functional in predicting thermochemical and kinetic properties [16,17], geometry optimizations and subsequent frequency analyses were conducted at the M06-2X/def2-TZVP level of theory—a method well-suited for modeling non-covalent interactions and electronic properties in systems like the present one [18]. The calculations incorporated Grimme’s D3 dispersion correction with the Becke-Johnson (BJ) damping function (keyword: em = GD3) to accurately describe dispersion forces [19]. Solvation effects in aqueous solution—relevant to AU’s applications—were simulated using the SMD solvation model based on the polarizable continuum model (PCM); the cavity was constructed with the GePol algorithm and a Lebedev grid (approx. 5.0 points/Å^2^) [20].

**Figure 2:**
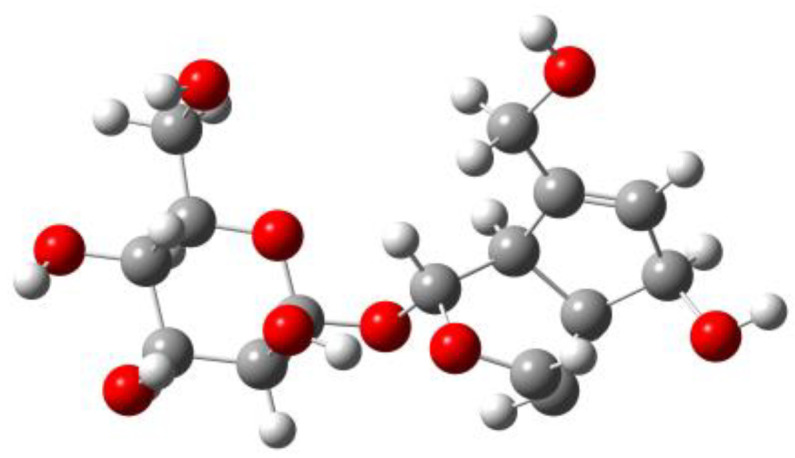
Molecular Structure Model of AU under the Optimization Conditions of M06-2X/def2-TZVP/scrf = (smd, solvent = water)/Grimme D3. White balls represent hydrogen atoms, gray balls represent carbon atoms, and red balls represent oxygen atoms.

Stringent convergence criteria were applied for the self-consistent field (SCF) procedure, with thresholds set to 1.0 × 10^−6^ a.u. for the energy change, 1.0 × 10^−6^ a.u. for the maximum density matrix change, and 1.0 × 10^−8^ a.u. for the root-mean-square density matrix change. Numerical integration of the DFT equations was performed using the FineGrid option. Frequency analysis confirmed the absence of imaginary frequencies, verifying that each optimized structure corresponds to a local minimum on the potential energy surface. And the final energy of the optimized aucubin structure (Figure 2) was calculated to be −1261.7531 Hartree.

Electronically, neutral AU molecules were treated using the restricted formalism, while radical intermediates generated from dehydrogenation or proton/electron transfer were treated using the unrestricted formalism. Optimized structures, spin densities, and frontier molecular orbitals were visualized using GaussView 6.0.16, and electrostatic potential (ESP) and natural bond orbital (NBO) charges were analyzed using Multiwfn 3.8.

### 3.2. Basis for Reaction Mechanism

Studies have shown that flavonoids and phenols exhibit antioxidant activity mainly through three mechanisms: HAT, SET-PT, and SPLET [21,22]. Although iridoid compounds possess diverse structural features, the AU molecule contains highly reactive functional groups, including hemiacetal-enol ether and ethylene oxide-olefin double bonds [23,24], which suggests that it may share similar antioxidant mechanisms. In this study, the interaction between AU and ^•^OH was investigated to determine which of the three potential mechanisms was most probable and dominant.

HAT mechanism: AU molecules (AU-OH) transfer hydrogen atoms to ^•^OH through the homolysis of the O-H bond to generate H_2_O and AU free radicals (AU-O^•^) (Equation (1)), and the activity is evaluated by BDE (AU-O-H) (Equation (2)).

AU-OH + ^•^OH → AU-O^•^ + H_2_O (1)

BDE = H(AU-O^•^) + H(H^•^) − H(AU-OH) (2)

SET-PT mechanism (two steps): (1) ^•^OH captures electrons from AU-OH to generate OH^−^ and AU-OH^•+^ (Equation (3)); (2) OH^−^ captures protons from AU-OH^•+^ to generate H_2_O and AU-O^•^ (Equation (4)). The activity is evaluated by IP (Equation (4)) and PDE (Equation (6)), respectively.

AU-OH + ^•^OH → OH^−^ + AU-OH^•+^ (3)

IP = H(e^−^) + H(AU-OH^•+^) − H(AU-OH) (4)

OH^−^ + AU-OH^•+^→ AU-O^•^ + H_2_O (5)

PDE = H(AU-O^•^) + H(H^+^) − H(AU-OH^•+^) (6)

SPLET mechanism (three steps): (1) AU-OH loses protons to generate AU-O^−^ and H^+^ (Equation (7)); (2) ^•^OH captures electrons from AU-O^−^ to generate AU-O^•^ and OH^−^ (Equation (9)); (3) OH^−^ combines with H^+^ to generate H_2_O (Equation (11)). The activity is evaluated by PA (Equation (8)) and ETH (Equation (10)), respectively.

AU-OH → AU-O^−^ + H^+^ (7)

PA = H(AU-O^−^) + H(H^+^) − H(AU-OH) (8)

AU-O^−^ + ^•^OH → AU-O^•^ + OH^−^ (9)

ETH = H(AU-O^•^) + H(e^−^) − H(AU-O^−^) (10)

OH^−^ + H^+^ → H_2_O (11)

Among them, H(AU-O^•^), H(AU-OH), H(AU-OH^•+^), H(AU-O^−^), and H(H^+^) represent the enthalpies of AU free radicals, AU molecules, AU cations, AU anions, and hydrogen ions.

## 4. Results and Discussion

### 4.1. Optimization of Molecular Structure

**Figure 3:**
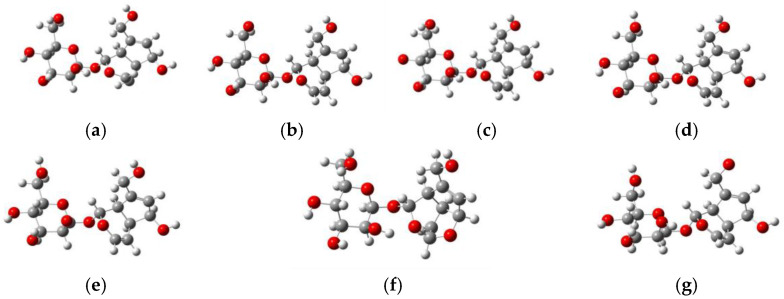
Optimized Molecular Structures of Aucubin and its Dehydrogenated Derivatives via Hydroxyl Radical Scission at Specific Sites (**a**) AU; (**b**) 4′-OH; (**c**) 5′-OH; (**d**) 6′-OH; (**e**) 7′-OH; (**f**) 6-OH; (**g**) 10-OH. White balls represent hydrogen atoms, gray balls represent carbon atoms, and red balls represent oxygen atoms.

Through structural optimization, the stable configurations of the aucubin molecule and its dehydrogenated derivatives—generated via homolytic cleavage of hydrogen from various hydroxyl groups—were obtained Figure 3. The results indicated that dehydrogenation at 4′-OH, 5′-OH, 7′-OH, and 10-OH sites did not induce significant change to the carbon skeletons of aucubin molecule. In contrast, dehydrogenation at 6-OH site resulted in the conversion of C3=C4 double bond into a single bond. The unpaired electron was transferred from the oxygen atom of 6-OH to C3, ultimately forming a new furanose ring structure with C3, C4, C5, and C6. The optimized structure exhibited the following geometric parameters: dihedral angles from 5.30° to 44.31° (range: 39.01°); bond angles from 101.01° to 108.05° (range: 7.04°); and bond lengths from 1.402 Å to 1.485 Å (range: 0.083 Å). The structure demonstrated high planarity and conjugation, which synergistically enhanced the stability of the resulting free radicals in conjunction with rings B and C. This conformational change can be rationalized by the bicyclic anomeric effect, epoxidation effect, and solvent electronic effect, as proposed by Gaweda et al. [25].

### 4.2. Frontier Molecular Orbital Analysis

**Figure 4:**
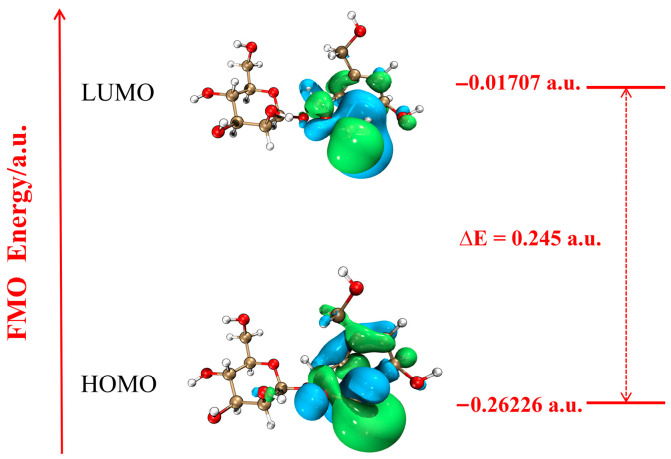
The FMO Schematic Diagram of the AU Molecule (Isosurface value of 0.05). White balls represent hydrogen atoms, gray balls represent carbon atoms, and red balls represent oxygen atoms.

Frontier molecular orbitals (FMO) provide insights into the underlying principles of chemical reaction mechanisms [26,27]. The calculation performed in this study (Figure 4) revealed that the FMO were primarily localized in the aglycone structure, with negligible distribution in the sugar ring structure, suggesting that the antioxidant reaction is likely to occur in the aglycone. However, whether the hydroxy groups on the sugar ring participate in reactions requires further investigation through charge distribution analyses. The highest occupied molecular orbital (HOMO) was mainly distributed in C5-C9 and C3=C4, and the lowest unoccupied molecular orbital (LUMO) was concentrated in C4-C5 and C3=C4, which was consistent with the structural optimization result of ring formation between C3 and C6 after the dehydrogenation of 6-OH (Figure 3f). Based on the M06-2X/def2-TZVP level, the HOMO-LUMO energy gap of AU was 0.245 a.u. (Figure 4). The low energy gap made it easy for HOMO electrons to be attacked by electrophilic reagents (^•^OH), which further confirmed the high reactivity of the aglycone hydroxy groups.

### 4.3. Calculation of Effective Charge Distribution

**Figure 5:**
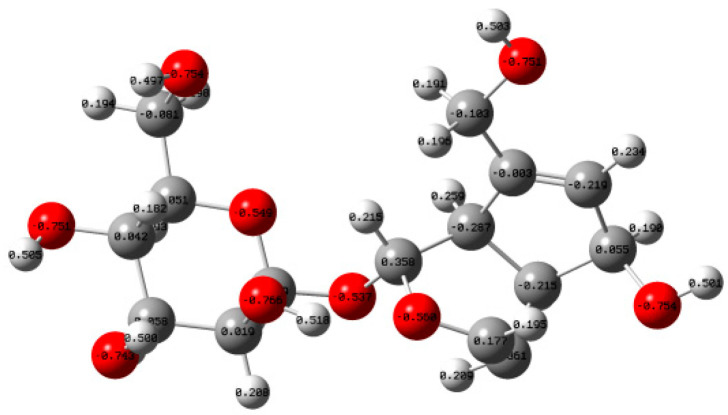
NPA Charge Distribution Diagram of Each Atom in AU Molecules. White balls represent hydrogen atoms, gray balls represent carbon atoms, and red balls represent oxygen atoms.

As shown in Figure 5, based on the natural bond orbital (NBO) theory, the atomic charges in the AU molecule were calculated using natural population analysis [28,29]. Table 2 presents the calculation results of the charge carried by the oxygen atom in each hydroxy group within the AU molecule.

It can be seen that compared with other hydroxy groups, the oxygen atom charge (−0.766 e) of the sugar ring 6′-OH was the lowest, and the hydrogen atom charge (0.518 e) was the highest, and the O-H bond dipole moment was the largest. This makes the sugar ring 6′-OH most susceptible to attack by electrophilic free radicals, readily undergoing homolytic dehydrogenation, confirming that the activity of the hydroxy groups on the sugar ring is non-negligible. This analytical approach is consistent with the method employed by Azad et al. for exploring intermolecular interactions [30].

### 4.4. Electrostatic Potential Analysis

**Figure 6:**
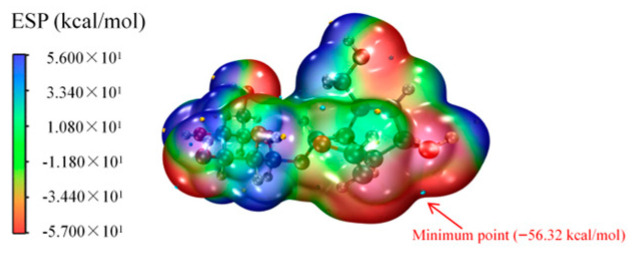
Schematic Diagram of the ESP of AU Molecules.

Electrostatic potential (ESP) can be used not only to analyze atomic interactions but also to identify reactive sites in chemical reactions [31,32]. By mapping the surface electrostatic potential (ESP) of AU molecules and searching for extreme points (Figure 6), it was found that the oxygen atom of 6-OH exhibited the lowest electrostatic potential (−56.32 kcal/mol), indicating that electrophilic hydroxyl radicals are more likely to attack this site. The strong negative ESP of the hydroxyl oxygen atom made it a priority target for hydroxyl radicals.

### 4.5. HAT Mechanism

In the HAT mechanism, the ease of releasing a hydrogen atom through O-H bond homolysis is measured by bond dissociation energy (BDE). A lower BDE indicates stronger antioxidant activity. As shown in the data in Table 3:

Priority of active sites: Whether in the gas-phase or aqueous solvent model, the 6-OH group shows the lowest BDE value (105.05 kJ/mol in the gas phase and 120.13 kJ/mol in the aqueous phase), followed by the 6′-OH group (191.33 kJ/mol in the gas phase and 189.39 kJ/mol in the aqueous phase). The BDE values are consistent with reports by Baj [33], Dao [34], and Thao [35], and align with conclusions from FMO, NBO, and ESP analyses.

Solvent effect: In the aqueous phase, the bond dissociation enthalpy (BDE) values for aucubin range from 120.13 kJ/mol to 282.80 kJ/mol. This indicates that aucubin is a significant antioxidant via the hydrogen atom transfer (HAT) pathway. All hydroxy groups in the aqueous solvent model have lower BDE values than those in the gas phase (e.g., the BDE of 4′-OH decreases from 449.41 kJ/mol to 272.56 kJ/mol), this effect may be attributed to the aqueous solvent environment. The lowest BDE of 6-OH matches earlier ESP analysis results.

### 4.6. SET-PT Mechanism

In the SET-PT mechanism, ionization potential (IP) characterizes electron transfer ability, while proton dissociation enthalpy (PDE) indicates proton transfer ability. The data in Table 4 demonstrates that low IP values in both models (498.91 kJ/mol in the gas phase and 314.36 kJ/mol in the aqueous phase), suggesting that AU easily loses electrons to form AU-OH^•+^. However, the high PDE values (912.90–1265.11 kJ/mol in the gas phase and 542.43–705.10 kJ/mol in the aqueous phase) indicate that AU-OH^•+^ is highly stable and difficult to release protons, hindering the second step of the reaction. Although IP is a key parameter in the SET-PT mechanism, the excessively high PDE creates a substantial energy barrier for electron transfer in the first step.

### 4.7. SPLET Mechanism

The feasibility of the SPLET mechanism is evaluated using proton affinity (PA) and electron transfer enthalpy (ETH). Table 5 shows that the PA value of the AU is extremely high (1523.43 kJ/mol in the gas phase and 708.57 kJ/mol in the aqueous phase), indicating strong basicity and high stability, which suggests that proton dissociation is very unfavorable in the first step and that the mechanism is unlikely to occur. Meanwhile, it can also be observed that only the ETH value of the 6-OH group in the gas phase is negative (−110.52 kJ/mol), confirming that the hydroxy group at this position releases heat during ETH. In contrast, the ETH values of hydroxy groups at other positions—whether in the gas phase or under the aqueous solvent model—are positive, indicating heat absorption. This implies that electron transfer in this mechanism requires external energy input.

In summary, the thermodynamic feasibility of the SPLET mechanism is significantly lower than that of the HAT mechanism.

**Table 5:** The PA Values and ETH Values of the SPLET Mechanism in Two Models.

Computational Model	Hydroxyl Position	PA (kJ/mol)	ETH (kJ/mol)
298.15 K/1.0 atm air	4′-OH	1445.92	311.36
	5′-OH	1493.55	271.57
	6′-OH	1416.47	82.72
	7′-OH	1400.75	338.43
	6-OH	1523.43	−110.52
	10-OH	1516.20	227.10
scrf = (smd, solvent = water)	4′-OH	699.07	310.78
	5′-OH	699.46	310.56
	6′-OH	426.73	499.95
	7′-OH	707.91	312.18
	6-OH	545.98	311.44
	10-OH	708.57	306.81

### 4.8. Gibbs Free Energy Calculation

According to studies by Sharma [36], Corciova [37], Amić [38], and others, the Gibbs free energy change (ΔG) serves as a key thermodynamic criterion for assessing whether a chemical reaction can proceed spontaneously under specific conditions and for comparing the propensity of different reactions to occur. The data for Gibbs free energy at each reaction step, presented in the tables, are accurate, as evidenced by the consistent total reaction Gibbs free energies across the three mechanisms in Table 6, Table 7 and Table 8. A comparison of Table 6 with Table 7 and Table 8 provides the following insights: Although the total ΔG values for the SET-PT and SPLET mechanisms are negative, the ΔG for the first step in both mechanisms is highly positive (383.48 kJ/mol for SET-PT in the gas phase and 1381.16 kJ/mol for SPLET in the gas phase). This suggests kinetic hindrance due to a high-energy transition state, which makes spontaneous progression difficult. The Gibbs free energy for subsequent steps is negative, implying the need for “energy coupling”—such as adding metal ion catalysts or using light energy—to quickly consume intermediates and drive the overall reaction. This aligns with research by Ostrowski et al., Cheng, Lai and Green [22,39,40], and offers guidance for optimizing the antioxidant reaction pathway in future studies.

### 4.9. Energy Changes in Different Models

Combining data from Table 3, Table 4 and Table 5, the energy requirements for the HAT mechanism (105.05–457.26 kJ/mol in the gas phase and 120.13–282.80 kJ/mol in the aqueous phase) are lower than those for the SET-PT mechanism (498.91 kJ/mol in the gas phase and 314.36 kJ/mol in the aqueous phase) and the SPLET mechanism (1400.75–1523.43 kJ/mol in the gas phase and 426.73–708.57 kJ/mol in the aqueous phase). Additionally, comparing Table 6, Table 7 and Table 8 reveals that only the Gibbs free energy change in the reaction via the HAT mechanism is negative (indicating a spontaneous reaction), while the Gibbs free energy change in the first step in the reactions via the SPLET and SET-PT mechanisms is positive (indicating non-spontaneous reactions). This confirms that the HAT mechanism proceeds preferentially.

As analyzed from Table 3 and Table 6, the 6-OH group in the HAT mechanism is most prone to cleavage and dehydrogenation (with the lowest BDE), followed by the 6′-OH group. For the same hydroxy group, the BDE value in the aqueous solvent model is lower than that in the gas phase, confirming that water molecules or polar environments are conducive to the cleavage and dehydrogenation of hydroxy groups, thereby enhancing antioxidant activity.

In the SET-PT and SPLET mechanisms, the energy parameters (IP, PDE, ETH) of the 6′-OH and 6-OH groups are superior to those of other hydroxy groups, indicating that these two sites are susceptible to microenvironmental disturbances and tend to switch to the HAT mechanism. This provides a scientific entry point for the structural design of multi-functional antioxidants.

## 5. Conclusions

Antioxidant Activity: AU exhibits significant hydroxyl radical (^•^OH) scavenging activity, with the scavenging rate increasing from 19.44% at 0.37 mmol/L to 47.04% at 5.89 mmol/L. At this concentration, its activity slightly surpasses that of ascorbic acid (45.14%) at the same concentration, highlighting its potential as a natural antioxidant.

Dominant Mechanism and Active Sites: Theoretical calculations indicate that hydrogen atom transfer (HAT) is the dominant mechanism for AU in scavenging ^•^OH. The key active sites are identified as 6-OH (aglycone structure) and 6′-OH (sugar ring structure). Their high reactivity is attributed to the high polarity of the O-H bond (maximum NPA charge difference), low bond dissociation energy (BDE) values (105.05 kJ/mol and 120.13 kJ/mol), the vulnerability of molecular orbitals to attack (HOMO-LUMO energy gap of 0.245 a.u.), and the stability provided by the conjugated structure. This study is the first to confirm the antioxidant activity of the hydroxy group in the sugar ring, overcoming the previous limitation of focusing solely on the aglycone structure.

Solvent Effect: Polar solvents, such as water, facilitate the cleavage of O-H bonds through intermolecular hydrogen bonding, reducing the BDE value for the HAT mechanism and significantly enhancing antioxidant activity. This finding provides theoretical support for the use of AU in aqueous-phase systems, such as in food and cosmetics.

This study offers molecular-level evidence for understanding the antioxidant mechanisms of iridoid compounds. The identified active sites and dominant reaction pathways provide valuable insights into the structural modification of AU, the development of targeted delivery systems, and the creation of multi-functional antioxidants.

## Figures and Tables

**Table 1:** Determination of Hydroxyl Radical Scavenging Rate.

Test Group	Concentration (mmol/L)	Absorbance (Ā_0_)	Absorbance (Ā_x_)	Scavenging Rate (%)
Positive control group	5.89	0.319	0.175	45.14
Sample group A	5.89	0.321	0.170	47.04
Sample group B	2.95	0.315	0.195	38.10
Sample group C	1.47	0.318	0.219	31.13
Sample group D	0.74	0.322	0.243	24.53
Sample group E	0.37	0.319	0.257	19.44

**Table 2:** Charges of Oxygen Atoms in Various Hydroxy Groups of AU Molecules.

Hydroxyl Position	Oxygen Atom Charge (e)	Hydrogen Atom Charge (e)
4′-OH	−0.751	0.505
5′-OH	−0.743	0.500
6′-OH	−0.766	0.518
7′-OH	−0.754	0.497
6-OH	−0.754	0.501
10-OH	−0.751	0.503

The position of each hydroxyl group, i.e., the serial number of the carbon atom it is attached to, refers to Figure 1.

**Table 3:** The BDE Values of AU Molecules in Two Models.

Computational Model	Hydroxyl Position	BDE (kJ/mol)
298.15 K/1.0 atm air	4′-OH	449.41
	5′-OH	457.26
	6′-OH	191.33
	7′-OH	431.32
	6-OH	105.05
	10-OH	435.43
scrf = (smd, solvent = water)	4′-OH	272.56
	5′-OH	272.73
	6′-OH	189.39
	7′-OH	282.80
	6-OH	120.13
	10-OH	278.09

**Table 4:** The IP Values and PDE Values of the SET-PT Mechanism in Two Models.

Computational Model	Hydroxyl Position	IP (kJ/mol)	PDE (kJ/mol)
298.15 K/1.0 atm air	4′-OH	498.91	1257.26
	5′-OH	498.91	1265.11
	6′-OH	498.91	999.17
	7′-OH	498.91	1239.17
	6-OH	498.91	912.90
	10-OH	498.91	1243.28
scrf = (smd, solvent = water)	4′-OH	314.36	694.86
	5′-OH	314.36	695.03
	6′-OH	314.36	611.69
	7′-OH	314.36	705.10
	6-OH	314.36	542.43
	10-OH	314.36	700.39

**Table 6:** Gibbs Free Energy of the HAT Mechanism in Two Models.

	Computational Model	298.15 K/1.0 atm Air Gibbs Free Energy Change, ΔG (kJ/mol)	scrf = (smd, solvent = water) Gibbs Free Energy Change, ΔG (kJ/mol)
Hydroxyl Position			
4′-OH		−40.60	−239.85
5′-OH		−33.52	−236.71
6′-OH		−301.53	−317.31
7′-OH		−57.73	−224.74
6-OH		−380.06	−379.31
10-OH		−55.28	−232.50

**Table 7:** Gibbs Free Energy of the SET-PT Mechanism in Two Models.

	Computational Model	298.15 K/1.0 atm Air Gibbs Free Energy Change, ΔG (kJ/mol)			scrf = (smd, solvent = water) Gibbs Free Energy Change, ΔG (kJ/mol)		
Hydroxyl Position		First Step	Second Step	Total Reaction	First Step	Second Step	Total Reaction
4′-OH		383.48	−424.09	−40.60	−206.51	−33.34	−239.85
5′-OH		383.48	−417.01	−33.52	−206.51	−30.20	−236.71
6′-OH		383.48	−685.01	−301.53	−206.51	−110.80	−317.31
7′-OH		383.48	−441.21	−57.73	−206.51	−18.23	−224.74
6-OH		383.48	−763.54	−380.06	−206.51	−172.80	−379.31
10-OH		383.48	−438.77	−55.28	−206.51	−25.98	−232.50

**Table 8:** Gibbs Free Energy of the SPLET Mechanism in Two Models.

	Computational Model	298.15 K/1.0 atm Air Gibbs Free Energy Change, ΔG (kJ/mol)				scrf = (smd, solvent = water) Gibbs Free Energy Change, ΔG (kJ/mol)			
Hydroxyl Position		First Step	Second Step	Third Step	Total Reaction	First Step	Second Step	Third Step	Total Reaction
4′-OH		1426.05	184.61	−1651.27	−40.60	668.66	−215.00	−693.51	−239.85
5′-OH		1460.20	157.55	−1651.27	−33.52	668.43	−211.63	−693.51	−236.71
6′-OH		1387.34	−37.60	−1651.27	−301.53	395.26	−19.06	−693.51	−317.31
7′-OH		1381.16	212.38	−1651.27	−57.73	677.21	−208.44	−693.51	−224.74
6-OH		1492.69	−221.47	−1651.27	−380.06	522.52	−208.32	−693.51	−379.31
10-OH		1487.60	108.39	−1651.27	−55.28	677.71	−216.70	−693.51	−232.50

## Data Availability

The data that support the findings of this study are available from the corresponding author upon reasonable request. The raw data supporting the findings of this study are available from the corresponding authors upon reasonable request.
